# 2-[3-Meth­oxy-5-(pyrimidin-2-yl)phen­yl]pyrimidine

**DOI:** 10.1107/S1600536813002663

**Published:** 2013-02-02

**Authors:** Jiena Hu, Yang Wu, Dengqing Zhang, Xianying Li, Wusong Jin

**Affiliations:** a College of Chemistry, Chemical Engineering and Biotechnology, Donghua University, 2999 North Renmin Road, Songjiang, Shanghai 201620, People’s Republic of China; b School of Environmental Science and Engineering, Donghua University, 2999 North Renmin Road, Songjiang, Shanghai 201620, People’s Republic of China

## Abstract

The title compound, C_15_H_12_N_4_O, was synthesized by a standard Suzuki cross-coupling reaction. The terminal pyrim­idine rings are rotated at dihedral angles of 12.06 (4) and −13.13 (4)° with respect to the central benzene ring. In the crystal, the mol­ecules are connected by two kinds of C—H⋯N hydrogen bonds, forming zigzag chains along the *c* axis. Weak π–π inter­actions between the benzene and one of the pyrimidine rings are also found and stack the mol­ecules along the *b* axis [centroid–centroid distance = 4.112 (3) Å].

## Related literature

For general background to the chemistry of tridentate NCN ligands, see: Pugh & Danopoulos (2007 ▶); Wu *et al.* (2009 ▶, 2012 ▶); Williams (2009 ▶); Wang *et al.* (2010 ▶). For the synthesis of the title compound, see: Avitia *et al.* (2011 ▶); Wakioka *et al.* (2010 ▶); Cardenas & Echavarren (1999 ▶).
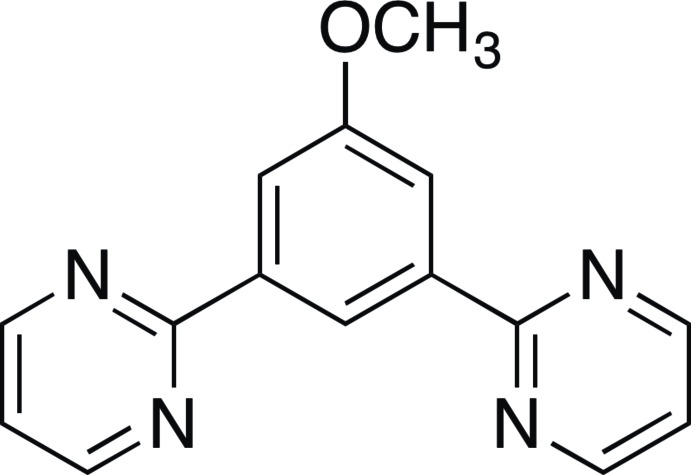


## Experimental

### 

#### Crystal data

C_15_H_12_N_4_O
*M* *_r_* = 264.29
Orthorhombic, 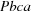

*a* = 11.3779 (8) Å
*b* = 8.0954 (6) Å
*c* = 27.3371 (18) Å
*V* = 2518.0 (3) Å^3^
*Z* = 8
Mo *K*α radiation
μ = 0.09 mm^−1^
*T* = 293 K
0.28 × 0.21 × 0.07 mm

#### Data collection

Bruker SMART CCD area-detector diffractometer
Absorption correction: multi-scan (*SADABS*; Bruker, 2000 ▶) *T*_min_ = 0.800, *T*_max_ = 1.000
14088 measured reflections
2461 independent reflections
2059 reflections with *I* > 2σ(*I*)
*R*_int_ = 0.030

#### Refinement

*R*[*F* ^2^ > 2σ(*F* ^2^)] = 0.044
*wR*(*F* ^2^) = 0.123
*S* = 1.04
2461 reflections
182 parameters
H-atom parameters constrained
Δρ_max_ = 0.16 e Å^−3^
Δρ_min_ = −0.21 e Å^−3^

### 

Data collection: *SMART* (Bruker, 2000 ▶); cell refinement: *SAINT* (Bruker, 2000 ▶); data reduction: *SAINT*; program(s) used to solve structure: *SHELXS97* (Sheldrick, 2008 ▶); program(s) used to refine structure: *SHELXL97* (Sheldrick, 2008 ▶); molecular graphics: *SHELXTL* (Sheldrick, 2008 ▶); software used to prepare material for publication: *SHELXTL*.

## Supplementary Material

Crystal structure: contains datablock(s) I, global. DOI: 10.1107/S1600536813002663/sj5297sup1.cif

Structure factors: contains datablock(s) I. DOI: 10.1107/S1600536813002663/sj5297Isup2.hkl

Supplementary material file. DOI: 10.1107/S1600536813002663/sj5297Isup3.cml

Additional supplementary materials:  crystallographic information; 3D view; checkCIF report

## Figures and Tables

**Table 1:** Hydrogen-bond geometry (Å, °)

*D*—H⋯*A*	*D*—H	H⋯*A*	*D*⋯*A*	*D*—H⋯*A*
C9—H9⋯N4^i^	0.93	2.69	3.597 (2)	166
C15—H15*B*⋯N3^ii^	0.96	2.69	3.547 (2)	149

Symmetry codes: (i) 
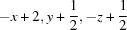
; (ii) 
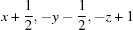
.

**Fig. 1.**
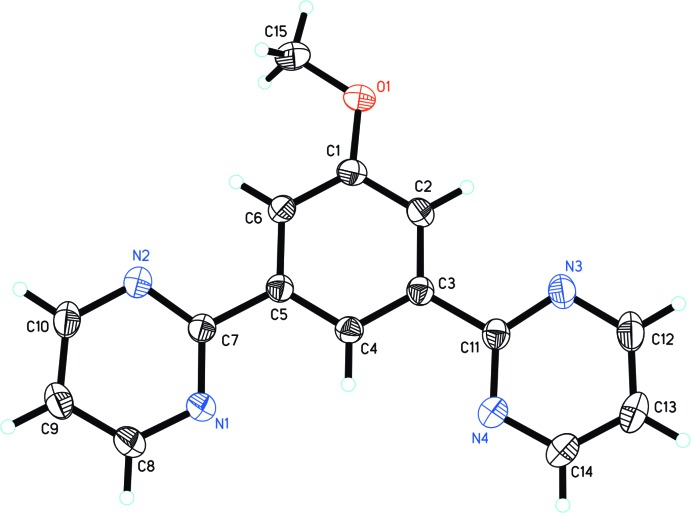
A perspective view of the title compound with displacement ellipsoids drawn at the 30% probability level.

**Fig. 2.**
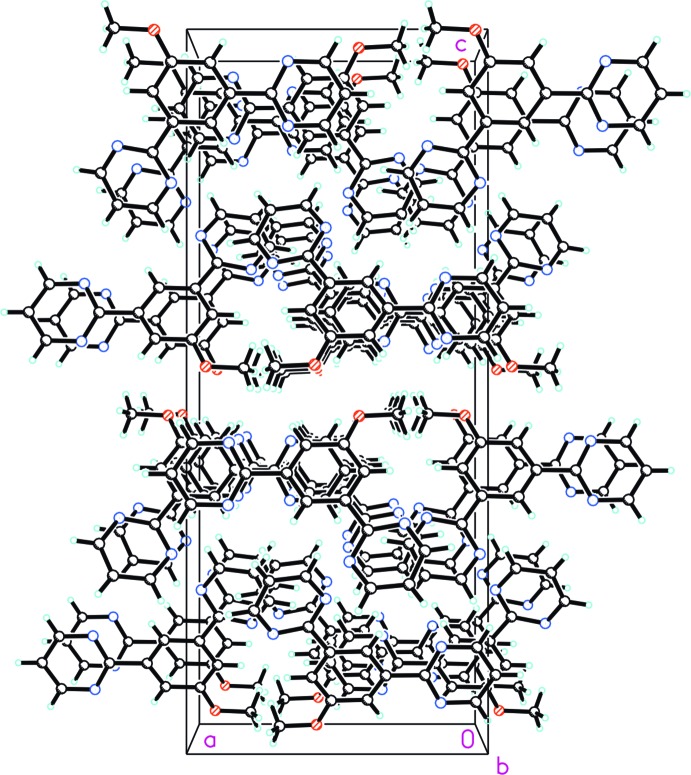
A view of the unit-cell contents of the title compound along the b axis.

## supplementary crystallographic information

### Comment

Pincer compounds have extensive applications in the coordination chemistry and material science (Pugh & Danopoulos, 2007). The most widely-used pincer ligand is terpyridine. Meanwhile, some tridentate NCN ligands with similar structures to terpyridine have also been reported (Williams, 2009; Wang *et al.*, 2010; Wu *et al.*, 2009). As part of our ongoing studies in this field (Wu *et al.*, 2012), we report here the crystal structure of a new pincer compound 1,3-di(pyrimin-2-yl)-5-methoxybenzene.

The molecular structure of the title compound is shown in Figure 1. The three aryl rings of the title compound are not coplanar, but dihedral angles of 12.06 (4)° and -13.13 (4)° are found between the terminal pyrimidine rings and central phenyl ring, respectively. In the crystal, intermolecular C9–H8···N4 and C15–H15B···N3 hydrogen bonds (Table 1) form zig-zag chains along *c*. The molecules stack along the *b* axis in such a way that the same pyrimidine-phenyl segment of two neighboring molecules overlap in opposite directions with a centroid-centroid distance of 4.112 (3) Å.

### Experimental

The compound was prepared using methods described in the literature (Avitia *et al.*, 2011; Wakioka *et al.*, 2010; Cardenas & Echavarren, 1999). A mixture of 1,3-bis(4,4,5,5-tetramethyl-1,3,2-dioxaborolan-2-yl)-5-methoxybenzene (600 mg, 1.67 mmol), 2-bromopyrimidine (800 mg, 5.03 mmol), K_2_CO_3_ aq (2.0 *M*, 8.40 ml) and Pd(PPh_3_)_4_ (390 mg, 0.251 mmol) in toluene (30 ml) was stirred at 110°C for 48 h under an argon atmosphere. After cooling to room temperature, the reaction mixture was poured into water, and extracted with CH_2_Cl_2_. The combined organic phase was washed with water and dried over anhydrous magnesium sulfate. After filtration and evaporation, the residue was purified with flash column chromatography (SiO_2_, eluted with CH_2_Cl_2_/petroleum ether = 1:5) to obtain the 1,3-di(pyrimidin-2-yl)-5-methoxybenzene as a white powder (353 mg) in 80% yield.^1^H NMR (400 MHz, CDCl_3_) δ 9.16 (s, 1H), 8.83 (d, *J* = 4.8 Hz, 4H), 8.14 (d, *J* = 1.5 Hz, 2H), 7.20 (t, *J* = 4.8 Hz, 2H), 3.99 (s, 3H). ^13^C NMR(100 MHz, CDCl_3_) δ 164.30, 160.51, 157.23, 139.36, 120.85, 119.32, 115.95, 55.74. MS(EI): m/*z*= 263.95. A single-crystal was obtained by slow evaporation of an acetonitrile solution of the title compound.

### Refinement

All H atoms were placed geometrically and treated as riding on their parent atoms with C—H = 0.96 (methyl) Å [U iso (H) = 1.5U eq (C)], and C—H = 0.93 (aromatic) Å [U iso (H) = 1.2U eq (C)].

### Crystal data

**Table:**

C_15_H_12_N_4_O	*F*(000) = 1104
*M**_r_* = 264.29	*D*_x_ = 1.394 Mg m^−^^3^
Orthorhombic, *P**b**c**a*	Mo *K*α radiation, λ = 0.71073 Å
Hall symbol: -P 2ac 2ab	Cell parameters from 4404 reflections
*a* = 11.3779 (8) Å	θ = 4.7–56.5°
*b* = 8.0954 (6) Å	µ = 0.09 mm^−^^1^
*c* = 27.3371 (18) Å	*T* = 293 K
*V* = 2518.0 (3) Å^3^	Prismatic, colourless
*Z* = 8	0.28 × 0.21 × 0.07 mm

### Data collection

**Table:**

Bruker SMART CCD area-detector diffractometer	2461 independent reflections
Radiation source: fine-focus sealed tube	2059 reflections with *I* > 2σ(*I*)
Graphite monochromator	*R*_int_ = 0.030
φ and ω scans	θ_max_ = 26.0°, θ_min_ = 1.5°
Absorption correction: multi-scan (*SADABS*; Bruker, 2000)	*h* = −14→12
*T*_min_ = 0.800, *T*_max_ = 1.000	*k* = −8→9
14088 measured reflections	*l* = −33→31

### Refinement

**Table:**

Refinement on *F*^2^	Primary atom site location: structure-invariant direct methods
Least-squares matrix: full	Secondary atom site location: difference Fourier map
*R*[*F*^2^ > 2σ(*F*^2^)] = 0.044	Hydrogen site location: inferred from neighbouring sites
*wR*(*F*^2^) = 0.123	H-atom parameters constrained
*S* = 1.04	*w* = 1/[σ^2^(*F*_o_^2^) + (0.0665*P*)^2^ + 0.4657*P*] where *P* = (*F*_o_^2^ + 2*F*_c_^2^)/3
2461 reflections	(Δ/σ)_max_ = 0.001
182 parameters	Δρ_max_ = 0.16 e Å^−^^3^
0 restraints	Δρ_min_ = −0.21 e Å^−^^3^

### Special details

**Table:**

Geometry. All e.s.d.'s (except the e.s.d. in the dihedral angle between two l.s. planes) are estimated using the full covariance matrix. The cell e.s.d.'s are taken into account individually in the estimation of e.s.d.'s in distances, angles and torsion angles; correlations between e.s.d.'s in cell parameters are only used when they are defined by crystal symmetry. An approximate (isotropic) treatment of cell e.s.d.'s is used for estimating e.s.d.'s involving l.s. planes.
Refinement. Refinement of *F*^2^ against ALL reflections. The weighted *R*-factor *wR* and goodness of fit *S* are based on *F*^2^, conventional *R*-factors *R* are based on *F*, with *F* set to zero for negative *F*^2^. The threshold expression of *F*^2^ > σ(*F*^2^) is used only for calculating *R*-factors(gt) *etc*. and is not relevant to the choice of reflections for refinement. *R*-factors based on *F*^2^ are statistically about twice as large as those based on *F*, and *R*- factors based on ALL data will be even larger.

### Fractional atomic coordinates and isotropic or equivalent isotropic displacement parameters (Å^2^)

**Table:**

	*x*	*y*	*z*	*U*_iso_*/*U*_eq_	
N1	1.03793 (11)	0.23473 (17)	0.28017 (4)	0.0556 (4)	
N2	1.20879 (9)	0.20037 (16)	0.32842 (4)	0.0508 (3)	
N3	0.65549 (10)	−0.04972 (17)	0.43132 (4)	0.0550 (3)	
N4	0.65653 (10)	0.05619 (16)	0.35048 (4)	0.0519 (3)	
O1	1.07030 (9)	−0.16817 (14)	0.46649 (3)	0.0572 (3)	
C1	1.02113 (12)	−0.07963 (17)	0.42928 (4)	0.0434 (3)	
C2	0.89954 (12)	−0.07835 (17)	0.42817 (5)	0.0444 (3)	
H2	0.8574	−0.1340	0.4522	0.053*	
C3	0.84023 (11)	0.00505 (16)	0.39159 (4)	0.0394 (3)	
C4	0.90336 (11)	0.08851 (16)	0.35561 (4)	0.0387 (3)	
H4	0.8639	0.1440	0.3308	0.046*	
C5	1.02519 (11)	0.08866 (16)	0.35693 (4)	0.0380 (3)	
C6	1.08424 (11)	0.00412 (16)	0.39390 (4)	0.0415 (3)	
H6	1.1660	0.0040	0.3948	0.050*	
C7	1.09451 (11)	0.17957 (16)	0.31955 (4)	0.0392 (3)	
C8	1.10177 (14)	0.3194 (2)	0.24815 (6)	0.0609 (4)	
H8	1.0644	0.3616	0.2206	0.073*	
C9	1.21922 (13)	0.3476 (2)	0.25355 (6)	0.0554 (4)	
H9	1.2626	0.4064	0.2306	0.067*	
C10	1.26891 (12)	0.2841 (2)	0.29482 (6)	0.0567 (4)	
H10	1.3490	0.3001	0.2998	0.068*	
C11	0.70953 (11)	0.00467 (17)	0.39113 (4)	0.0417 (3)	
C12	0.53848 (13)	−0.0495 (2)	0.43016 (6)	0.0627 (5)	
H12	0.4977	−0.0875	0.4574	0.075*	
C13	0.47587 (13)	0.0040 (2)	0.39070 (6)	0.0609 (4)	
H13	0.3941	0.0056	0.3907	0.073*	
C14	0.53930 (13)	0.0549 (2)	0.35126 (6)	0.0589 (4)	
H14	0.4991	0.0905	0.3236	0.071*	
C15	1.19460 (14)	−0.1760 (2)	0.46883 (6)	0.0639 (5)	
H15A	1.2246	−0.2221	0.4390	0.096*	
H15B	1.2177	−0.2443	0.4959	0.096*	
H15C	1.2258	−0.0668	0.4732	0.096*	

### Atomic displacement parameters (Å^2^)

**Table:**

	*U*^11^	*U*^22^	*U*^33^	*U*^12^	*U*^13^	*U*^23^
N1	0.0475 (6)	0.0735 (9)	0.0457 (6)	−0.0099 (6)	−0.0043 (5)	0.0165 (6)
N2	0.0395 (6)	0.0582 (8)	0.0548 (7)	−0.0046 (5)	−0.0003 (5)	0.0092 (6)
N3	0.0450 (7)	0.0736 (9)	0.0466 (7)	−0.0109 (6)	0.0072 (5)	−0.0010 (6)
N4	0.0403 (6)	0.0610 (8)	0.0542 (7)	0.0031 (5)	0.0002 (5)	0.0046 (6)
O1	0.0531 (6)	0.0696 (7)	0.0491 (6)	0.0027 (5)	−0.0065 (4)	0.0201 (5)
C1	0.0468 (7)	0.0460 (8)	0.0375 (6)	0.0020 (6)	−0.0027 (5)	0.0030 (6)
C2	0.0463 (8)	0.0490 (8)	0.0378 (6)	−0.0033 (6)	0.0050 (5)	0.0046 (6)
C3	0.0387 (7)	0.0411 (7)	0.0383 (6)	−0.0012 (5)	0.0021 (5)	−0.0037 (5)
C4	0.0395 (7)	0.0415 (7)	0.0352 (6)	0.0008 (5)	−0.0013 (5)	0.0001 (5)
C5	0.0387 (7)	0.0395 (7)	0.0357 (6)	−0.0010 (5)	0.0012 (5)	−0.0027 (5)
C6	0.0359 (6)	0.0472 (8)	0.0414 (7)	0.0001 (5)	−0.0014 (5)	−0.0003 (6)
C7	0.0380 (6)	0.0410 (7)	0.0385 (6)	−0.0010 (5)	0.0006 (5)	−0.0021 (5)
C8	0.0611 (9)	0.0768 (11)	0.0449 (8)	−0.0123 (8)	−0.0027 (7)	0.0170 (8)
C9	0.0587 (9)	0.0581 (9)	0.0495 (8)	−0.0128 (7)	0.0122 (6)	0.0053 (7)
C10	0.0397 (7)	0.0626 (10)	0.0677 (10)	−0.0083 (7)	0.0078 (6)	0.0072 (8)
C11	0.0400 (7)	0.0425 (7)	0.0426 (7)	−0.0017 (5)	0.0036 (5)	−0.0050 (6)
C12	0.0465 (8)	0.0834 (12)	0.0582 (9)	−0.0140 (8)	0.0140 (7)	−0.0097 (8)
C13	0.0356 (7)	0.0702 (11)	0.0768 (11)	−0.0021 (7)	0.0063 (7)	−0.0170 (9)
C14	0.0416 (8)	0.0652 (10)	0.0699 (10)	0.0051 (7)	−0.0055 (7)	−0.0001 (8)
C15	0.0553 (9)	0.0735 (11)	0.0628 (9)	0.0086 (8)	−0.0128 (7)	0.0178 (8)

### Geometric parameters (Å, º)

**Table:**

N1—C8	1.3280 (18)	C4—H4	0.9300
N1—C7	1.3316 (17)	C5—C6	1.3934 (17)
N2—C10	1.3310 (18)	C5—C7	1.4859 (17)
N2—C7	1.3333 (16)	C6—H6	0.9300
N3—C12	1.3317 (19)	C8—C9	1.364 (2)
N3—C11	1.3338 (16)	C8—H8	0.9300
N4—C11	1.3311 (17)	C9—C10	1.363 (2)
N4—C14	1.3340 (18)	C9—H9	0.9300
O1—C1	1.3643 (15)	C10—H10	0.9300
O1—C15	1.4171 (18)	C12—C13	1.363 (2)
C1—C6	1.3821 (18)	C12—H12	0.9300
C1—C2	1.3838 (18)	C13—C14	1.361 (2)
C2—C3	1.3826 (18)	C13—H13	0.9300
C2—H2	0.9300	C14—H14	0.9300
C3—C4	1.3928 (17)	C15—H15A	0.9600
C3—C11	1.4872 (18)	C15—H15B	0.9600
C4—C5	1.3867 (17)	C15—H15C	0.9600
			
C8—N1—C7	116.21 (12)	N1—C8—H8	118.3
C10—N2—C7	116.10 (12)	C9—C8—H8	118.3
C12—N3—C11	116.16 (13)	C10—C9—C8	115.66 (13)
C11—N4—C14	115.93 (12)	C10—C9—H9	122.2
C1—O1—C15	117.79 (11)	C8—C9—H9	122.2
O1—C1—C6	124.49 (12)	N2—C10—C9	123.39 (13)
O1—C1—C2	115.47 (11)	N2—C10—H10	118.3
C6—C1—C2	120.03 (11)	C9—C10—H10	118.3
C3—C2—C1	120.49 (12)	N4—C11—N3	125.60 (12)
C3—C2—H2	119.8	N4—C11—C3	117.35 (11)
C1—C2—H2	119.8	N3—C11—C3	117.04 (11)
C2—C3—C4	119.74 (12)	N3—C12—C13	122.78 (14)
C2—C3—C11	119.55 (11)	N3—C12—H12	118.6
C4—C3—C11	120.71 (11)	C13—C12—H12	118.6
C5—C4—C3	119.83 (11)	C14—C13—C12	116.48 (14)
C5—C4—H4	120.1	C14—C13—H13	121.8
C3—C4—H4	120.1	C12—C13—H13	121.8
C4—C5—C6	120.04 (11)	N4—C14—C13	123.02 (15)
C4—C5—C7	120.87 (11)	N4—C14—H14	118.5
C6—C5—C7	119.09 (11)	C13—C14—H14	118.5
C1—C6—C5	119.87 (12)	O1—C15—H15A	109.5
C1—C6—H6	120.1	O1—C15—H15B	109.5
C5—C6—H6	120.1	H15A—C15—H15B	109.5
N1—C7—N2	125.18 (12)	O1—C15—H15C	109.5
N1—C7—C5	117.74 (11)	H15A—C15—H15C	109.5
N2—C7—C5	117.07 (11)	H15B—C15—H15C	109.5
N1—C8—C9	123.44 (14)		
			
C15—O1—C1—C6	−0.5 (2)	C6—C5—C7—N1	168.58 (12)
C15—O1—C1—C2	178.92 (13)	C4—C5—C7—N2	167.50 (12)
O1—C1—C2—C3	−178.88 (12)	C6—C5—C7—N2	−11.89 (18)
C6—C1—C2—C3	0.6 (2)	C7—N1—C8—C9	1.4 (2)
C1—C2—C3—C4	−0.05 (19)	N1—C8—C9—C10	−0.6 (3)
C1—C2—C3—C11	179.97 (12)	C7—N2—C10—C9	0.5 (2)
C2—C3—C4—C5	−0.56 (18)	C8—C9—C10—N2	−0.4 (2)
C11—C3—C4—C5	179.41 (11)	C14—N4—C11—N3	1.3 (2)
C3—C4—C5—C6	0.63 (18)	C14—N4—C11—C3	−179.78 (13)
C3—C4—C5—C7	−178.76 (11)	C12—N3—C11—N4	−0.9 (2)
O1—C1—C6—C5	178.91 (12)	C12—N3—C11—C3	−179.84 (13)
C2—C1—C6—C5	−0.52 (19)	C2—C3—C11—N4	−166.49 (13)
C4—C5—C6—C1	−0.10 (18)	C4—C3—C11—N4	13.54 (18)
C7—C5—C6—C1	179.31 (12)	C2—C3—C11—N3	12.55 (18)
C8—N1—C7—N2	−1.3 (2)	C4—C3—C11—N3	−167.43 (12)
C8—N1—C7—C5	178.20 (13)	C11—N3—C12—C13	−0.5 (2)
C10—N2—C7—N1	0.4 (2)	N3—C12—C13—C14	1.4 (3)
C10—N2—C7—C5	−179.09 (13)	C11—N4—C14—C13	−0.2 (2)
C4—C5—C7—N1	−12.03 (18)	C12—C13—C14—N4	−1.0 (2)

### Hydrogen-bond geometry (Å, º)

**Table:**

*D*—H···*A*	*D*—H	H···*A*	*D*···*A*	*D*—H···*A*
C9—H9···N4^i^	0.93	2.69	3.597 (2)	166
C15—H15*B*···N3^ii^	0.96	2.69	3.547 (2)	149

Symmetry codes: (i) −*x*+2, *y*+1/2, −*z*+1/2; (ii) *x*+1/2, −*y*−1/2, −*z*+1.
